# Grading and Sorting of Grape Berries Using Visible-Near Infrared Spectroscopy on the Basis of Multiple Inner Quality Parameters

**DOI:** 10.3390/s19112600

**Published:** 2019-06-07

**Authors:** Hui Xiao, Li Feng, Dajie Song, Kang Tu, Jing Peng, Leiqing Pan

**Affiliations:** 1 College of Food Science and Technology, Nanjing Agriculture University, Nanjing 210095, China; 2015108046@njau.edu.cn (H.X.); fengli@njau.edu.cn (L.F.); kangtu@njau.edu.cn (K.T.); jpeng@njau.edu.cn (J.P.); 2 School of computer and Information Engineering, Chuzhou University, Chuzhou 239000, China; songdajie@chzu.edu.cn

**Keywords:** grape, grading and sorting, total phenolic compounds, total soluble solid content, partial least squares regression

## Abstract

The potential of visible-near infrared (vis/NIR) spectroscopy (400 nm to 1100 nm) for classification of grape berries on the basis of multi inner quality parameters was investigated. Stored *Vitis vinifera* L. cv. Manicure Finger and *Vitis vinifera L*. cv. Ugni Blanc grape berries were separated into three classes based on the distribution of total soluble solid content (*SSC*) and total phenolic compounds (*TP*). Partial least squares regression (PLS) was applied to predict the quality parameters, including color space CIELAB, *SSC*, and *TP*. The prediction results showed that the vis/NIR spectrum correlated with the *SSC* and *TP* present in the intact grape berries with determination coefficient of prediction (*R*_P_^2^) in the range of 0.735 to 0.823. Next, the vis/NIR spectrum was used to distinguish between berries with different *SSC* and *TP* concentrations using partial least squares discrimination analysis (PLS-DA) with >77% accuracy. This study provides a method to identify stored grape quality classes based on the spectroscopy and distributions of multiple inner quality parameters.

## 1. Introduction

Grapes are berry fruits with thin peels and high moisture and sugar content, and are more sensitive to storage and transportation conditions than other types of fruits. As photosynthesis is halted after fruit harvest, respiration becomes the main metabolic process during storage and transportation. This catabolism results in the consumption of organic matter in fruit, affecting the edible quality and nutritional value of post-harvest grapes and considerably reducing their commodity value. Garcia [1] observed that frozen storage affects grape quality parameters, including total soluble solid content (*SSC*), pH, titratable acidity, total anthocyanin content, and total phenolic compounds (*TP*). However, studies on evaluation of grape berry quality during cold storage are limited. Improving the cold storage resistance of grapes is an important aspect of postharvest preservation of grape fruit, while the non-destructive monitoring of grape quality during storage and the rapid determination of the freshness of grapes are new points to be considered for effective and rational arrangement of the stock and reduction in storage loss after harvest [2].

According to the brochure of International Standardization of Fruits and Vegetables—Table Grape, published by the Organization for Economic Co-operation and Development (OECD), commercial table grapes are divided into three quality types, namely, superior quality, good quality, and marketable quality [3]. This standard focuses more on the entire bunch quality, including the total *SSC*, appearance, shape, color, and weight. However, Parpinello [4] pointed out that total *SSC* varies considerably within each grape cluster, and Nelson [5] reported that this variability may exceed 6%. As berries in a cluster face different direction, they are exposed to varying degrees of sunlight, and hence the pigment and *TP* content of the berries are also unequal. Thus, it is reasonable to monitor grape bunch quality from a berry point of view. For any specific sample, the external and internal qualities deteriorate during storage. However, the changes in external and internal qualities are asynchronous for different samples, and the specific numerical range for each quality parameter in specific varieties are not standardized. Thus, grading of postharvest and stored grape berries is challenging. In this study, we proposed a method of grading berries numerically based on their inner quality and the whole sample set distribution during short cold storage. Based on external quality, all berry samples remained marketable after storage Previously, Glasbey [6] has used size distribution information for potato tuber grading schemes. 

The traditional method is not only time-consuming and expensive, but is also damaging for the grape berries. Sample analysis is a time-consuming process, which unavoidably alters sample quality, especially close to the grape harvest season. Spectroscopic techniques, such as visible (vis), near infrared (NIR), mid infrared, and hyperspectral spectroscopy are more non-destructive and rapid than the traditional physical and chemical methods. Vis/NIR and NIR spectroscopy have the advantages of simple instrumentation and universality. Therefore, they are currently used in agriculture, food, petrochemical, and pharmaceutical industries. The vis/NIR technology has been extensively used to determine the chemical components in agricultural products, including soluble solid, water, protein, starch, lipid, and cellulose content, and grade the quality class and contamination levels of these samples [7,8,9,10]. In grape industry, Giovenzana [11] applied a handheld optic vis/NIR spectrometer to estimate the *SSC* and *TP* concentrations and Beghi [12] identified the ripeness of grape berries during ripening process. However, as we mentioned before, studies on using the vis/NIR technology to evaluate the grape berry quality during cold storage are limited. This is the first study to identify stored grape quality classes based on its multiple inner quality parameters combined with spectroscopy, which will assist in improving the economics of the grape and wine industry.

As the name suggests, vis/NIR spectroscopy consists of visible and near-infrared spectroscopy. The wavelength range used in visible spectroscopy is 380 nm to 760 nm, and sample color is related to absorption of visible light. When a sample surface is irradiated, electrons absorb visible light of a particular wavelength and transition to a higher energy level, which manifests as color. NIR radiation is a type of electromagnetic wave with wavelength between the visible and mid infrared regions of the spectrum. The energy of the photon in the NIR range is lower than that in the visible range and that required for electrons to escape from outer orbitals of alkali metals. Therefore, the information provided by the NIR spectrum is different from that obtained due to electronic transition in the visible light spectrum, although the absorption bands of structural components such as O-H, N-H, C-H, and other hydrogen-bearing groups, which are related to overtone bands and combination of several stretch-bend vibration modes, are similar [13]. Overall, vis/NIR spectroscopy can provide valuable information regarding grape quality and freshness.

Therefore, this study aims to (1) define the quality classes of grape berries based on the distributions of total *SSC* and *TP* and (2) distinguish berries of different classes using the vis/NIR spectra.

## 2. Materials and Methods

### 2.1. Samples Preparation

In this study, *Vitis vinifera* L. cv. Manicure Finger and *Vitis vinifera L*. cv. Ugni Blanc were used. The 60 clusters of each cultivar were collected in Nanjing Eight Diagrams Vineyard (32°09′59.75″ N; 118°49′29.82″ E, Nanjing, China) after ripening; all the clusters were stored at 4 °C in the presence of 85% to 95% relative humidity in freshness packets for 25 days. The sampling process was conducted every 5 days (0 d, 5 d, 10 d, 15 d, 20 d, and 25 d), and 10 clusters of each cultivar were collected each time, and three berries were randomly picked from the top, middle, and bottom of each cluster. All collected berries were free of visual defects.

### 2.2. Vis/NIR Spectroscopy Collection

The reflectance spectrum of each intact sample was obtained from 32 scans using a fiber optic vis/NIR system. The system consisted of a computer (Surface pro 3, Microsoft Corporation, New York, NY, USA), a vis/NIR fiber optic spectrometer (FX2000, Shanghai Ideaoptics Corporation, Shanghai, China) which uses holographic concave diffractive grating combined with charge-coupled component to obtain the spectra covering the range of 400 nm to 1100 nm, a quartz optical fiber, a halogen light source (HL 2000, Shanghai Ideaoptics Corporation, Shanghai, China) and a detection platform [14].

### 2.3. Physical and Chemical Analyses

#### 2.3.1. CIELAB

The CIELAB color space values were determined using a digital hand-held spectrophotometer (Ci6X, X-rite, Grand Rapids, MI, USA). The factor L* represents “lightness”, with values ranging from 0 to 100, factor a* represents color change from magenta to green, and b* represents color change from yellow to blue [15].

#### 2.3.2. SSC

According to the China Standard [16] for *SSC* measurement in fruit, after removing the peel of grape berries, extracting juice from the flesh of each sample was measured using a digital hand-held “pocket” refractometer (PAL-1, ATAGO CO., LTD., Tokyo, Japan), and was expressed as a percentage with an accuracy of 0.1% according to the refractometer’s specification. 

#### 2.3.3. TP

Each of stored grape berries was homogenized in liquid nitrogen using a batch mill (A 11 B S025, IKA, Staufen, Germany). Then, 0.2 g of the ground sample of each berry was macerated in 8 mL hydrochloric acid solution (10 mL/L HCl: 960 mL/L ethanol = 17:3 v/v) at 40 °C for 40 min under ultrasonication. The mixture was then centrifuged for 5 min at 13,710 g, and 0.1 mL of the supernatants were diluted with 4 mL HCl solution. Finally, *TP* content was determined by measuring the absorbance at 280 nm using a UV spectrophotometer (UV 1800, Shimadzu Corporation, Kyoto, Japan). Gallic acid monohydrate (in the range of 2.0 mg/L to 20.0 mg/L) was applied to generate a calibration curve and *TP* was expressed as concentration of gallic acid of fresh grape sample [17]. 

### 2.4. Grading of Stored Grape Berries

Considering that the ripening condition of each berry is different, and the quality change is asynchronous during the storage process, grading berries according to storage time is unreasonable. Therefore, grading based on the inner quality parameters appears appropriate. The data regarding total *SSC* and *TP* content were subjected to descriptive analysis to test the normal distribution of the data using the IBM SPSS Statistics 22 software (IBM Inc., New York, NY, USA). Then, the data was used to produce a frequency histogram and a Gaussian curve fitting. Berry classes were determined from the mean value (*μ*) and standard deviation (*σ*) of the fitted Gaussian equations.

The berries were divided into four classes based on *SSC* and *TP* distribution. The fourth class (IV) is characterized by *μ + σ* to +∞, the third class (III) by *μ + σ* to *μ*, the second class (II) by *μ − σ* to *μ*, and the first class (I) by *0* to *μ − σ*. Then, the *SSC*-based and *TP*-based divisional results were combined to divide the berries into three groups using the following chart (Table 1).

### 2.5. Determination and Discrimination Based on vis/NIR Spectra

The vis/NIR spectroscopy data were preprocessed using moving average smoothing (MS), the standard normal variate (SNV) [18], and mean normalization. The samples were divided into calibration and prediction sets by 3:1 for each class. The determination models were built using partial least squares (PLS) method and the discrimination models using partial least squares discrimination analysis (PLS-DA). Leave-one-out cross-validation (LOOCV) was used during model establishments. The preprocessing and model establishments were generated with MATLAB 2018b (The Mathworks, Natick, MA, USA). 

## 3. Results

### 3.1. Statistical Results of Quality Parameters

The statistical results of the reference parameters are shown in Table 2. For stored Manicure Finger berries, the L*, a*, and b* values were in the range of 34.82 to 49.50, 2.07 to 11.03, and 2.80 to 15.43, respectively; the average L*, a*, and b* values were 42.26, 5.94, and 9.41, respectively. For Ugni Blanc berries in cold storage, the L*, a*, and b* values were in the range of 37.21 to 44.85, −3.05 to −0.54 and 8.94 to 17.09, respectively, and the average values were 42.12, −1.93, and 12.61, respectively.

The *SSC* of Manicure Finger ranged from 13.93% to 19.01% and the mean value was 16.57%; the SSC of Ugni Blanc was in the range of 14.68% to 17.31% and the mean value was 15.81%. Spayd [19] observed that the *SSC* was unchanged after 24 h of frozen storage. Cynkar and co-workers [20,21] showed that the *SSC* did not change during frozen storage for 1 week, 2 weeks, 3 weeks, and 10 weeks, and for 1 month, 3 months, 6 months, and 12 months.

The phenolic compounds in Manicure Finger and Ugni Blanc varied considerably. This may be caused by the increase in activity of polyphenol oxidase, which oxidizes phenol compounds to quinone compounds and causes browning of the peel of grape berries; the a* value increases as the color of the peel darkens.

### 3.2. Grading of Stored Grape Berries

**Figure 1:**
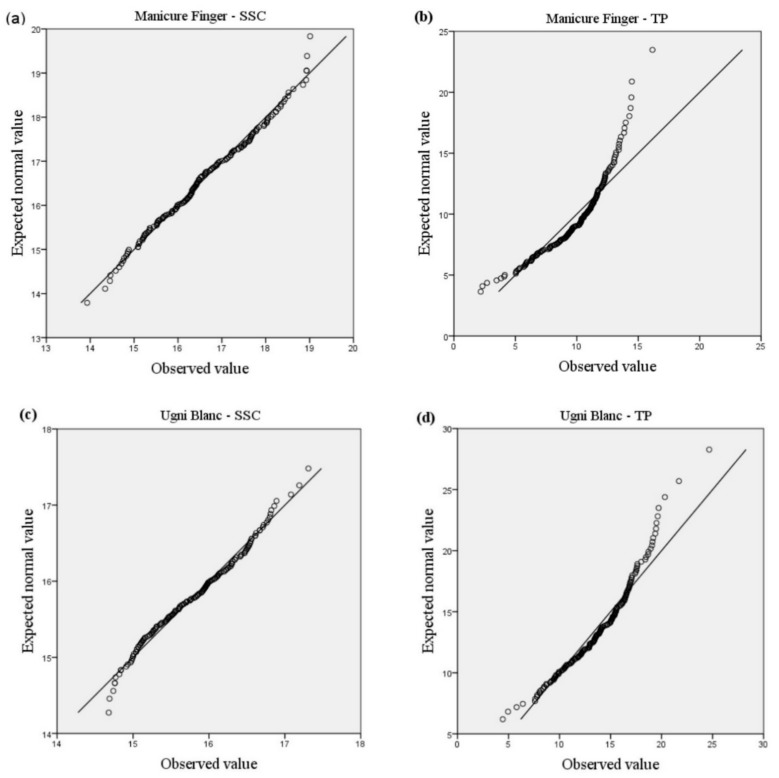
Normal probability plots for the distributions of total soluble solid content and total phenolic compounds in two grape varieties. SSC: soluble solid content; TP: total phenolic compounds. (**a**) expected and observed values of SSC in Manicure Finger; (**b**) expected and observed values of TP in Manicure Finger; (**c**) expected and observed values of SSC in Ugni Blanc; (**d**) expected and observed values of TP in Ugni Blanc).

The normal probability plots for the distribution of total *SSC* and *TP* compounds are shown in Figure 1. When few large and small values are ignored, all the plots appear fairly straight and the points lie close to a straight line in each plot, indicating that the distributions of total *SSC* and *TP* compounds of each variety are normal.

**Figure 2:**
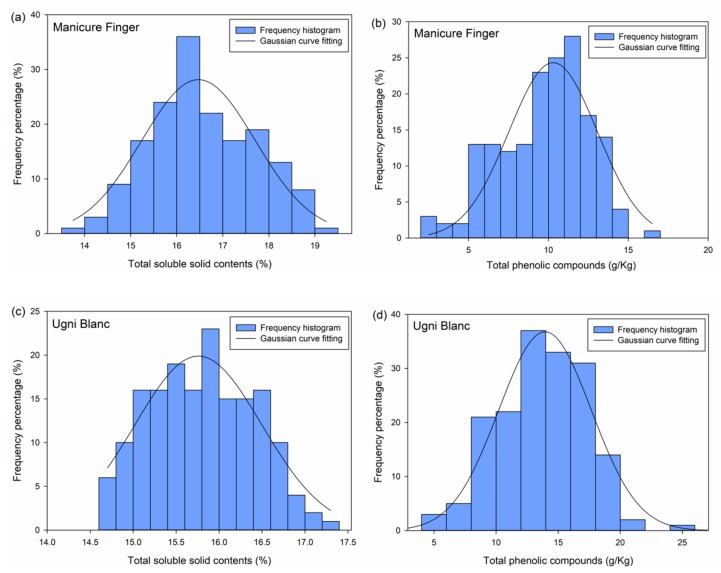
Frequency histograms and Gaussian curve fitting for total soluble solid content and total phenolic compounds. ((**a**), frequency percentage of SSC in Manicure Finger; (**b**), frequency percentage of TP in Manicure Finger; (**c**), frequency percentage of SSC in Ugni Blanc; (**d**), frequency percentage of TP in Ugni Blanc).

The frequency histogram of each parameter of each variety was plotted (Figure 2). All the histograms appear symmetric and unimodal, which is in accordance with the character of normal distribution. Hence, these plots indicated the normal distribution of each parameter of each variety. Based on the frequency results, the Gaussian curve fitting was performed on each parameter dataset as shown in Figure 2. The Gaussian equations are:

(1) $$f=28.14\times e^{-\frac{{\left(x-16.47\right)}^{2}}{6.04}}$$

(2) $$f=24.33\times e^{-\frac{{\left(x-10.2\right)}^{2}}{15.4}}$$

(3) $$f=19.89\times e^{-\frac{{\left(x-15.76\right)}^{2}}{1.09}}$$

(4) $$f=36.77\times e^{-\frac{{\left(x-13.94\right)}^{2}}{27.81}}$$

The correlation coefficients (*R*) were 0.93, 0.91, 0.94, and 0.97 for *SSC* and *TP* of Manicure Finger and Ugni Blanc, respectively. According to the probability density function (5) of normal distribution, the *μ* and *σ* value of each data set can also be computed. Thus, the *μ* and *σ* values can be assumed to be the mean value and standard deviation, respectively, when the dataset is large. The fitting values are close to the statistical results mentioned in Section 3.1.

(5) $$f=\frac{1}{\sigma \sqrt{2\pi }}e^{-\frac{{\left(x-\mu \right)}^{2}}{2\sigma^{2}}}$$

Next, grading was performed based on the fitted *μ* and *σ* values. The classes based on *SSC* distribution for Manicure Finger were as follows: class I (0% to 15.24%), class II (14.01% to 16.47%), class III (16.47% to 17.7%), and class IV (17.7% to +∞%). The classes based on *TP* distribution for Manicure Finger were as follows: class I (0 g/kg to 7.52 g/kg), class II (7.52 g/kg to 10.29 g/kg), class III (10.29 g/kg to 13.06 g/kg), and class IV (13.06 g/kg to +∞ g/kg). The classes based on *SSC* distribution for Ugni Blanc were as follows: class I (0% to 15.02%), class II (15.02% to 15.76%), class III (15.76% to 16.50%), and class IV (16.50% to +∞ %). The classes based on *TP* distribution for Ugni Blanc were as follows: class I (0 g/kg to 10.21 g/kg), class II (10.21 g/kg to 13.94 g/kg), class III (13.94 g/kg to 17.63 g/kg), and class IV (17.63 g/kg to +∞ g/kg). The berries were divided into three classes after combining the *SSC* grading results with *TP* grading results for each variety. For Manicure Finger berries, there were a total of 58 berries in class 1, a total of 70 berries in class 2, and a total of 44 berries in class 3; for Ugni Blanc, there were a total of 35 berries in class 1, a total of 91 berries in class 2, and a total of 43 berries in class 3.

### 3.3. Vis/NIR Spectral Features of Grape Berries

**Figure 3:**
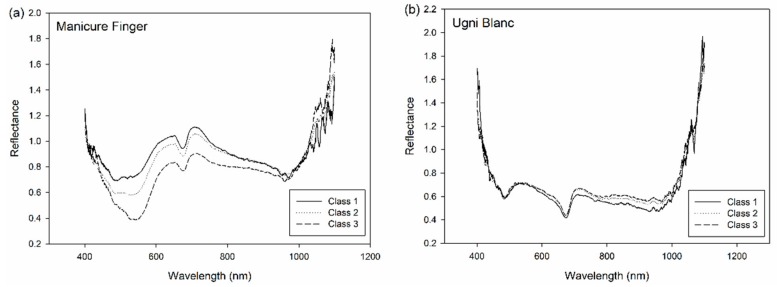
Mean reflectance spectra of ’Manicure Finger’ (**a**) and ’Ugni Blanc’ (**b**) in different classes.

The preprocessed reflectance spectra of Manicure Finger and Ugni Blanc are shown in Figure 3. The main differences in the spectral features were observed in the range of 400 nm to 760 nm, which is related to the absorbance of visible light by berries peels. As the two selected varieties showed different peel color, the features of the spectral peaks varied between these two varieties. Manicure Finger with red peel color absorbed green color around the range of 500 nm to 570 nm which is green light, and showed strong reflectance in the range of 600 nm to 700 nm which is red light. On the contrary, the white variety, Ugni Blanc, showed strong absorbance in the range of 450 nm to 500 nm (which is blue light) and 600 nm to 700 nm, and strong reflectance in the range of 500 nm to 570 nm which is green light, as the Ugni Blanc peel is green in color [22]. In the NIR range, a small peak can be observed around 950 nm to 970 nm both for ‘Manicure Finger’ and ‘Ugni Blanc’, which assigned to -OH second overtone [23]. 

Interestingly, the spectral difference among different classes were observed in the range of 400 nm to 1000 nm for Manicure Finger, while for Ugni Blanc, spectral differences were not evident in the range of 400 nm to 700 nm, and relatively minor changes were observed in the range of 700 nm to 1000 nm. These differences demonstrated that the berries in different classes may can be distinguished based on their spectral characteristics.

### 3.4. Determination of Quality Parameters Using vis/NIR Spectra

The partial least squares regression results are shown in Table 3. For the regression of color space values of Manicure Finger, the prediction for a* was the best among the three color parameters with the highest ratio of standard error of performance to standard deviation (*RPD*) of 2.191, determination coefficient of prediction (*R*_P_^2^) of 0.724, and root mean squares of prediction (*RMSEP*) of 1.324. The b* value was also predicted, with higher determination coefficient of calibration (*R*_C_^2^) and *R*_P_^2^ of 0.829 and 0.816, respectively, although *RPD* was lower than that of a*. In contrast, the prediction for b* was better than that of a*, with higher *R*^2^ and *RPD*. The *RMSE* values were not compared as they are for different parameters. The L* value prediction was not satisfactory, which might be because the vis/NIR spectra did not provide sufficient information regarding lightness.

The prediction of *SSC* for Manicure Finger was better than that for Ugni Blanc as the model for Manicure Finger was associated with slightly higher *R*^2^ and lower *RMSE* both for the calibration set and prediction set than that for Ugni Blanc; in addition, it showed higher *RPD* of 1.435. 

Similar observation was made for the Ugni Blanc berries. *R*_C_^2^ was 0.851 and *R*_P_^2^ was 0.823 for Manicure Finger prediction, which were higher than those for Ugni Blanc, with predictions of 0.811 and 0.735 for *R*_C_^2^ and *R*_P_^2^, respectively. The *RPD* of Manicure Finger was considerably higher than that of Ugni Blanc. 

With the exception of L*, the *R*^2^ values of all models were higher than 0.600, especially for *SSC* and *TP* prediction of two varieties, indicating that the models were applicable to some extents.

### 3.5. Distinguishing Berries Based on the vis/NIR Spectra

The results of PLS-DA are shown in Table 4. For calibration set of Manicure Finger berries, five, five, and three samples were misidentified in classes 1, 2, and 3, respectively. Thus, the distinguishing accuracies were 88.4%, 90.2%, and 90.6% for classes 1, 2, and 3, respectively, for the calibration set. When the constructed model was used to predict berries in the prediction set, three, four, and one samples were mistaken for the three respective classes, and the discrimination accuracies were 80.0%, 77.8%, and 90.9%, respectively.

For Ugni Blanc, two samples were misidentified in class 1 of the calibration set. Seven and two berries were misidentified in classes 2 and 3. The identification accuracies were 92.3%, 89.7%, and 93.8%, respectively. For the prediction set of Ugni Blanc, the accuracies were slightly lower than those of the calibration set, while two, five, and two samples were misidentified in the three classes (1, 2, and 3), respectively. 

**Figure 4:**
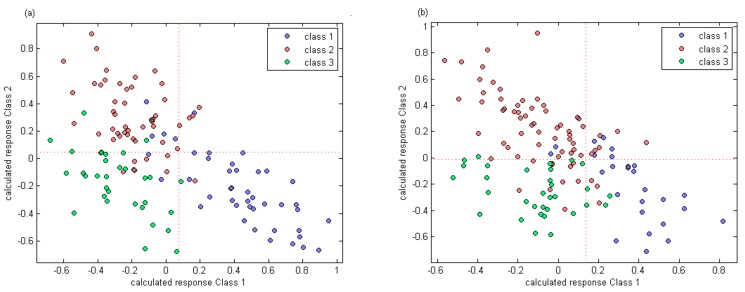
Calculated response of three classes using partial least squares discrimination analysis. (**a**) the calculated response of PLS-DA for Manicure Finger; (**b**) the calculated response of PLS-DA for Ugni Blanc)

The calculated response of three classes using partial least squares discrimination analysis for two varieties are shown in Figure 4.

## 4. Discussion

In this study, we proposed a method of grading grape berries using vis/NIR spectra, which can define the quality classes of grape berries based on the distributions of inner quality parameters such as *SSC* and *TP*. 

Cozzolino [24] reviewed the vis/NIR studies in grape and wine industry and observed that 900 nm to 1050 nm, 300 nm to 1160 nm, and 650 nm to 1100 nm in vis-short wavelength NIR (400 nm to 1100 nm) were commonly related to the prediction of total *SSC* in grapes, and a standard error of calibration (*SEC*) range of 0.5% to 3.9% must be achieved. Our prediction results and the spectral features of whole grapes in our study are in accordance with those of Cozzolino [25]. However, the prediction results of *SSC* and *TP* were worse than those of models constructed using developing and ripening berries of the same variety reported by Xiao [14]. This is possibly because a wide range of samples provide more information required for establishing a model.

As sugars and phenolic compounds are used as primary quantitative parameters of quality, their concentrations should be determined, and the freshness of stored fruit must be estimated according to these parameters [26]. Considering the complexity associated with the determination of quality parameters, berry sorting and grading is a big issue for fresh and stored fruits. Sorting and grading can not only ensure that the products satisfy different levels of consumer expectations but can also reduce losses by preventing cross-contamination between good and damaged fruits. The traditional sorting and grading systems involve use of molds with specific holes on a conveyor belt that sort fruits by size, and manually based on color, shape, and external defects. 

Nondestructive methods, including vis/NIR spectroscopy, X-ray tomography, and machine vision are widely used in agricultural production as they can rapidly detect physical and chemical qualities. Kondon [27] described several fruit and vegetable grading systems for orange, eggplant, and leek based on automation technologies and pointed out that machine vision systems are most widely used as exterior qualities are commonly the key quality features used for grading. However, because of the increasing demand for high quality products worldwide, especially in developed countries, the inner qualities of fruits, including sugar contents, acidity, and nutrient contents should also be included as inspection parameters. For the grape industry, Lafontaine [28] pointed out that berry sorting can assist in improving wine quality, as wine flavor depends significantly on sugar and phenolic compound content. They constructed an experimental sorter to sort grape berries according to their maturity/sugar concentration, for which maturity is the indication of phenolic compound content. This sorter works by first floating the berries in different salt solutions to determine total soluble solids levels, followed by vis/NIR spectrum to determine maturity stages. Negara [29] assessed the LDA, KNN-C, SVM PCR, and PLSR to classify grape berries into high sugar and low sugar concentration groups, and observed that vis/NIR spectra were better for classification than the conventional NIR spectra, and that PLS can be used successfully for this purpose. Compared to their results, our models distinguished berries based on both total *SSC* and *TP* compounds by determining the relationship between vis/NIR spectrum and chemical compounds, and achieved relatively promising results. This can be used in grape industry for improving the efficiency of sorting and grading grapes.

## 5. Conclusions

This study defined grape berry classes based on the distribution of inner quality parameters, namely, total *SCC* and *TP* compounds, and also built chemometric models to distinguish between berries from different classes. Results showed that the range of accuracies for all discrimination models were in the range of 77% to 94%. Considering that efficient sorting and labor saving are two important parts of modern fruit and vegetable industry, more nondestructive technologies would be tested and utilized on-line, and how to improve the discrimination accuracy and how to take more quality requirements into consideration may be some of the new research directions. 

## Figures and Tables

**Table 1:** Quality classes of stored berries based on total soluble solid content and total phenolic compounds.

	*SSC*	I	II	III	IV
*TP*					
I		1	1	1	1
II		1	2	2	2
III		1	2	3	3
IV		1	2	3	3

**Table 2:** Statistical results of each parameter for two varieties.

Variety	Parameter	Min	Max	Mean	SD
Manicure Finger	L*	34.82	49.50	42.26	3.49
	a*	2.07	11.03	5.94	1.97
	b*	2.80	15.43	9.41	2.76
	*SSC*/%	13.93	19.01	16.57	1.13
	*TP*/(g/kg)	2.18	16.16	9.72	2.75
Ugni Blanc	L*	37.21	44.85	42.12	1.26
	a*	−3.05	−0.54	−1.93	0.73
	b*	8.94	17.09	12.61	1.48
	*SSC*/%	14.68	17.31	15.81	0.60
	*TP*/(g/kg)	4.43	24.67	13.73	3.50

SD: Standard deviation.

**Table 3:** PLS regression for *SSC*, color, and *TP* of Manicure Finger and Ungi Blanc berries based on full band spectra.

Variety	Parameter	*R*_C_^2^	*RMSEC*	*R*_P_^2^	*RMSEP*	*RPD*
Manicure Finger	L*	0.624	6.033	0.537	7.315	0.615
	a*	0.781	0.890	0.724	1.324	2.191
	b*	0.829	2.787	0.816	2.887	1.212
	*SSC*/%	0.833	0.764	0.799	0.976	1.435
	*TP*/(g/kg)	0.851	0.114	0.823	0.164	1.830
Ugni Blanc	L*	0.610	4.729	0.589	5.432	0.700
	a*	0.686	0.834	0.624	0.967	0.620
	b*	0.819	3.653	0.702	4.022	0.795
	*SSC*/%)	0.813	1.157	0.793	1.575	0.546
	*TP*/(g/kg)	0.811	0.157	0.735	0.185	0.544

*R*_C_^2^: Determination coefficient of calibration; *R*_P_^2^: Determination coefficient of prediction; *RMSEC*: root mean square of calibration; *RMSEP*: root mean square of prediction; *RPD*: ratio of standard error of performance to standard deviation.

**Table 4:** Discrimination accuracies of two varieties from different classes using partial least squares discrimination analysis.

Variety	Set	Class	Class I	Class II	Class III	Accuracy%
Manicure Finger	Calibration	Class I	38	4	1	88.4
		Class II	1	46	4	90.2
		Class III	1	2	29	90.6
	Prediction	Class I	12	3	0	80.0
		Class II	4	14	0	77.8
		Class III	0	1	10	90.9
Ugni Blanc	Calibration	Class I	24	2	0	92.3
		Class II	2	61	5	89.7
		Class III	2	0	30	93.8
	Prediction	Class I	7	1	1	77.8
		Class II	4	18	1	78.3
		Class III	0	2	9	81.8
